# Supplementary material for: Constructing phase boundary in AgNbO3 antiferroelectrics: pathway simultaneously achieving high energy density and efficiency
Source: Nat Commun. 2020 Sep 24;11:4824. doi: 10.1038/s41467-020-18665-5 (PMC7515927; doi:10.1038/s41467-020-18665-5)
Supplement: Supplementary file 1 — Supplementary Information [file 41467_2020_18665_MOESM1_ESM.pdf]

Supplementary Information

Constructing phase boundary in $AgNbO_3$ antiferroelectrics: pathway simultaneously achieving high energy density and efficiency

Luo et al.

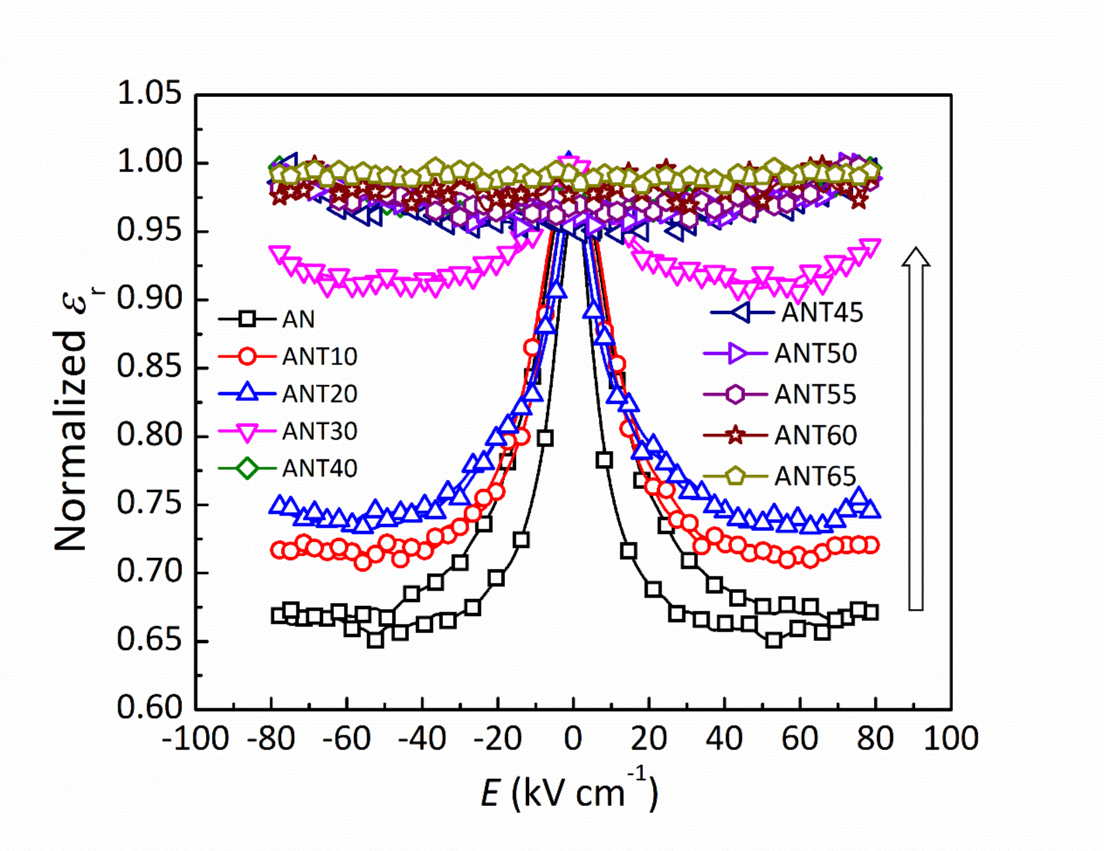

Supplementary Fig. 1 Electric field dependence of normalized dielectric permittivity. The variation of dielectric permittivity decreases more obvious with the increase of electric field for low Ta doped compositions, which is more flattened for the high Ta concentration compositions. This indicates the antiferroelectricity is enhanced with increase of Ta content.

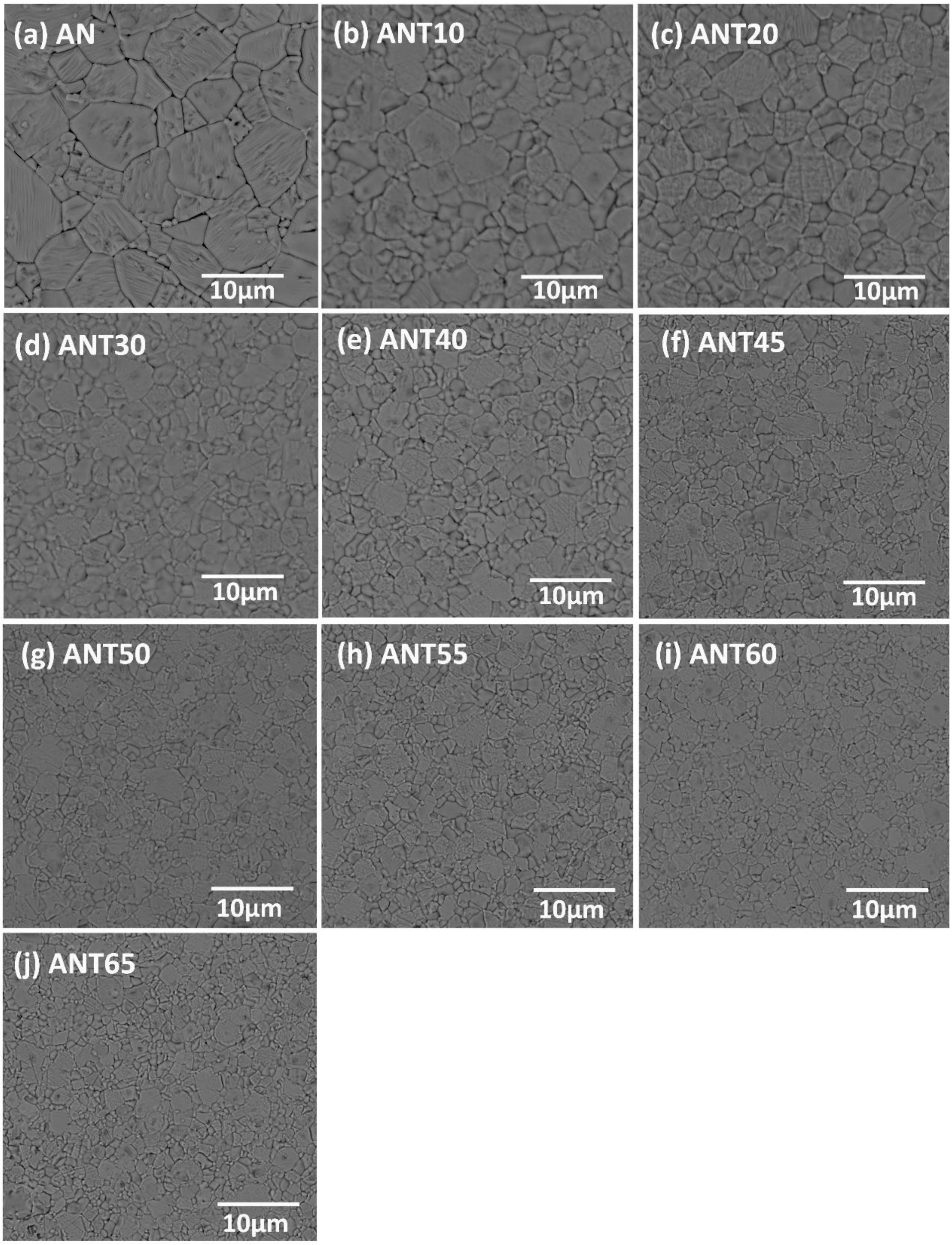

Supplementary Fig. 2 SEM micrographs of the as-sintered ANT*x* ceramics. (a)AN, (b)ANT10, (c)ANT20, (d)ANT20, (e)ANT40, (f)ANT45, (g)ANT50, (h)ANT55, (i)ANT60, (j)ANT65.

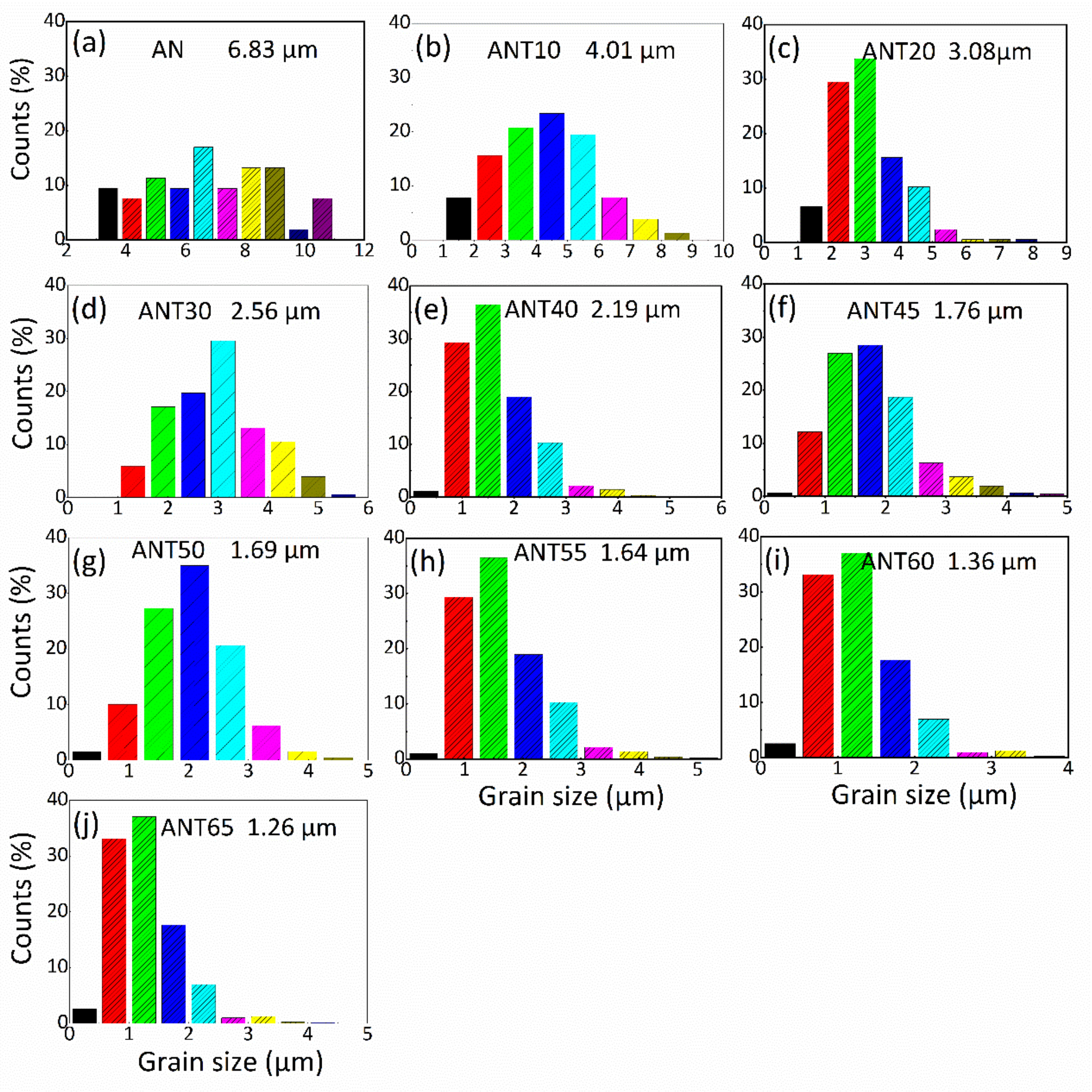

Supplementary Fig. 3 Grain size distribution of the as-sintered ANT*x* ceramics. (a)AN, (b)ANT10, (c)ANT20, (d)ANT20, (e)ANT40, (f)ANT45, (g)ANT50, (h)ANT55, (i)ANT60, (j)ANT65. The grain size distribution obeys Gaussian distribution, which is much narrower with the increase of Ta content.

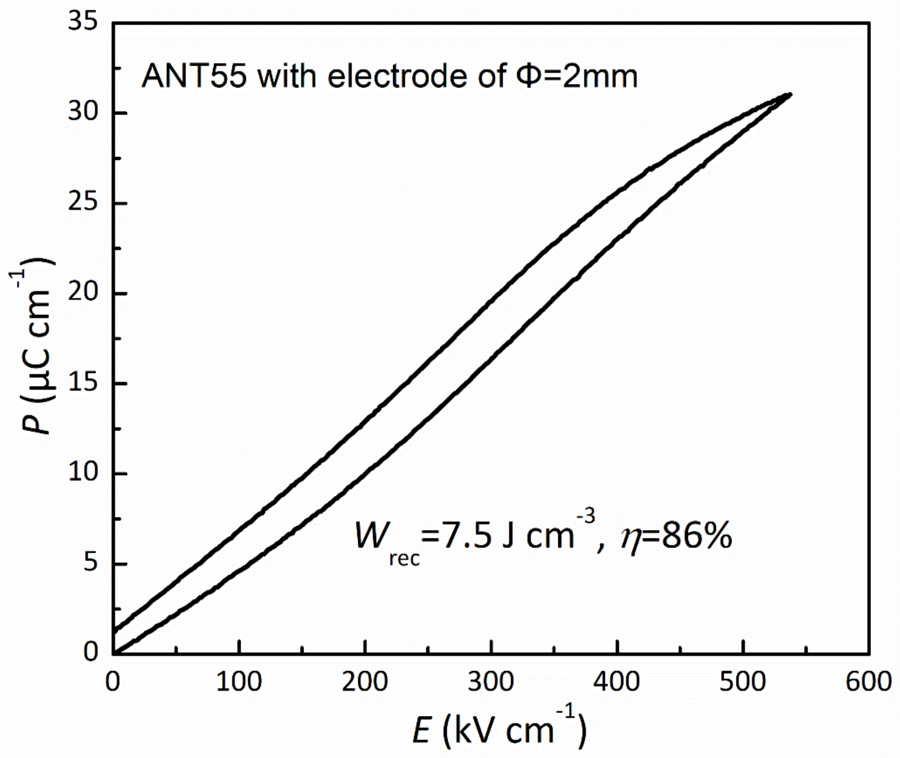

Supplementary Fig. 4 *P*-*E* loops for ANT55 sample, for which the sample was polished to ~80 um in thickness and coated by gold electrode with a diameter of 2mm.

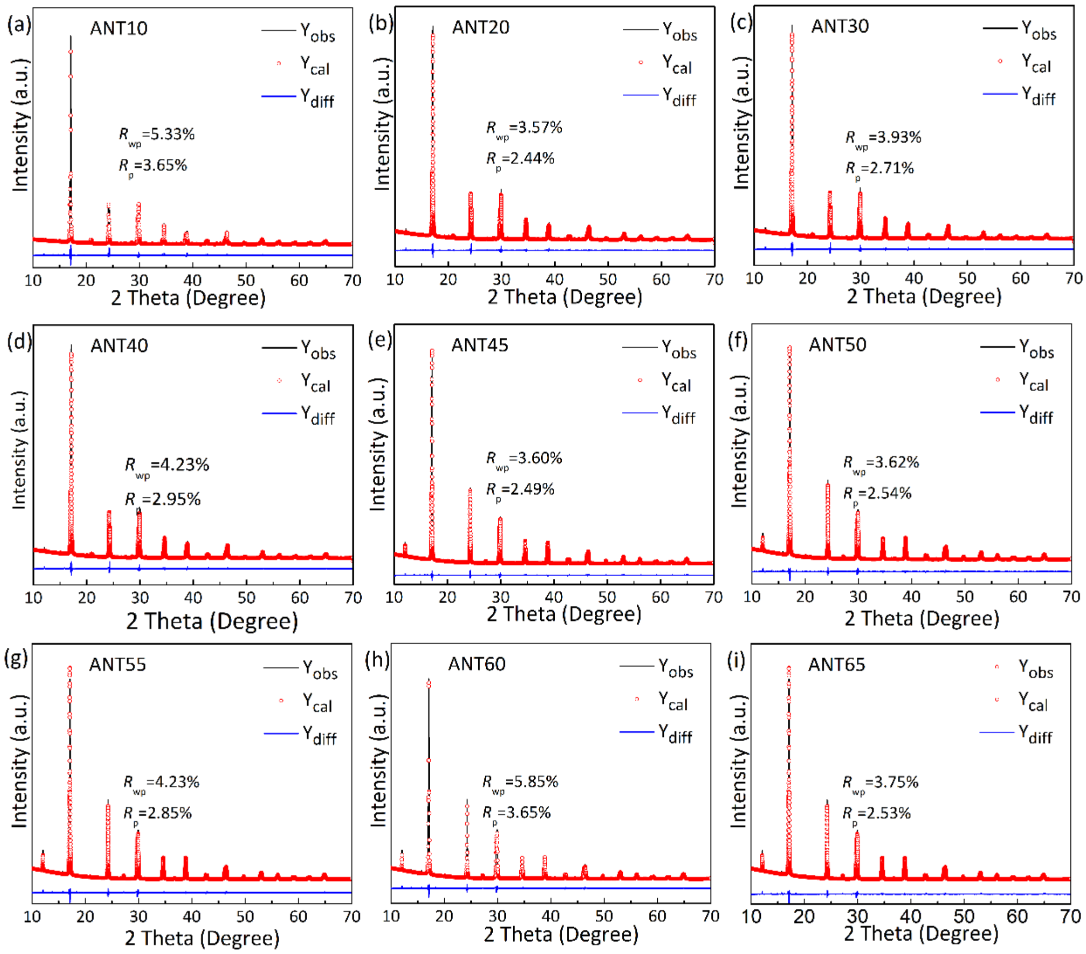

Supplementary Fig. 5 Rietveld refinement on synchrotron x-ray patterns of as-prepared ANT*x* ceramic powders, based on Pbcm space group. (a)ANT10, (b)ANT20, (c)ANT30, (d)ANT40, (e)ANT45, (f)ANT50, (g)ANT55, (h)ANT60, (i)ANT65. The low reliability factor values indicate the structural model is valid and the refinement results fit well with the experimental data.

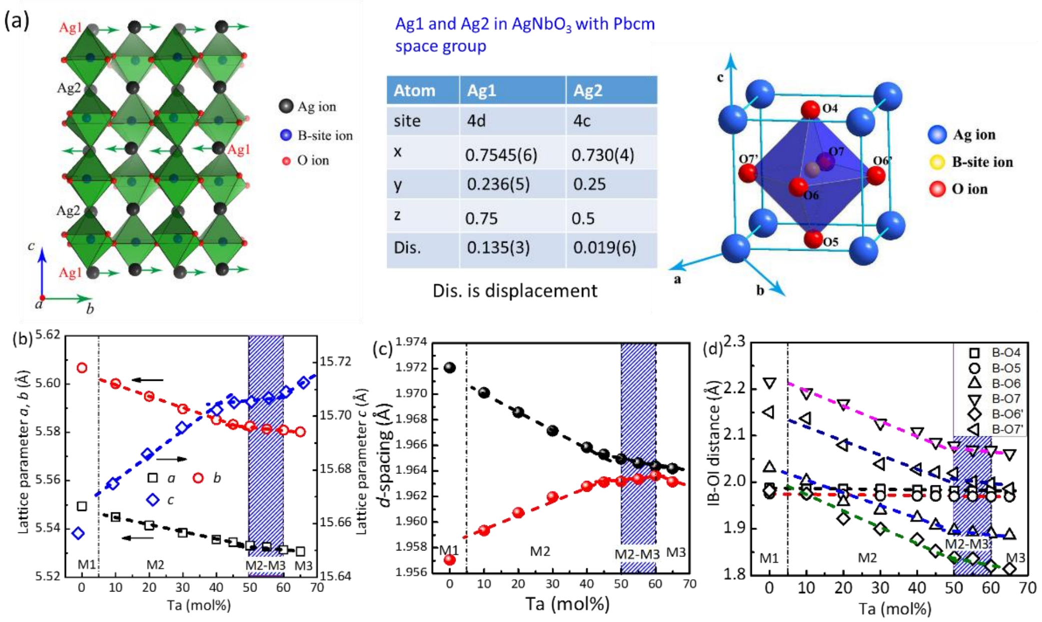

Supplementary Fig. 6 The structure and refined crystal parameters evolution as a function of Ta for the as-prepared ANT*x* ceramics. (a) The structure and parameters of Ag1, Ag2 and O ions in $AgNbO_3$ with Pbcm space group. Rietveld lattice parameters of (b) *a*, *b* and *c*, (c) the (220) and (008) d-spacing, and (d) |B-O| distance as a function of Ta for the as-prepared ANT*x* ceramics.

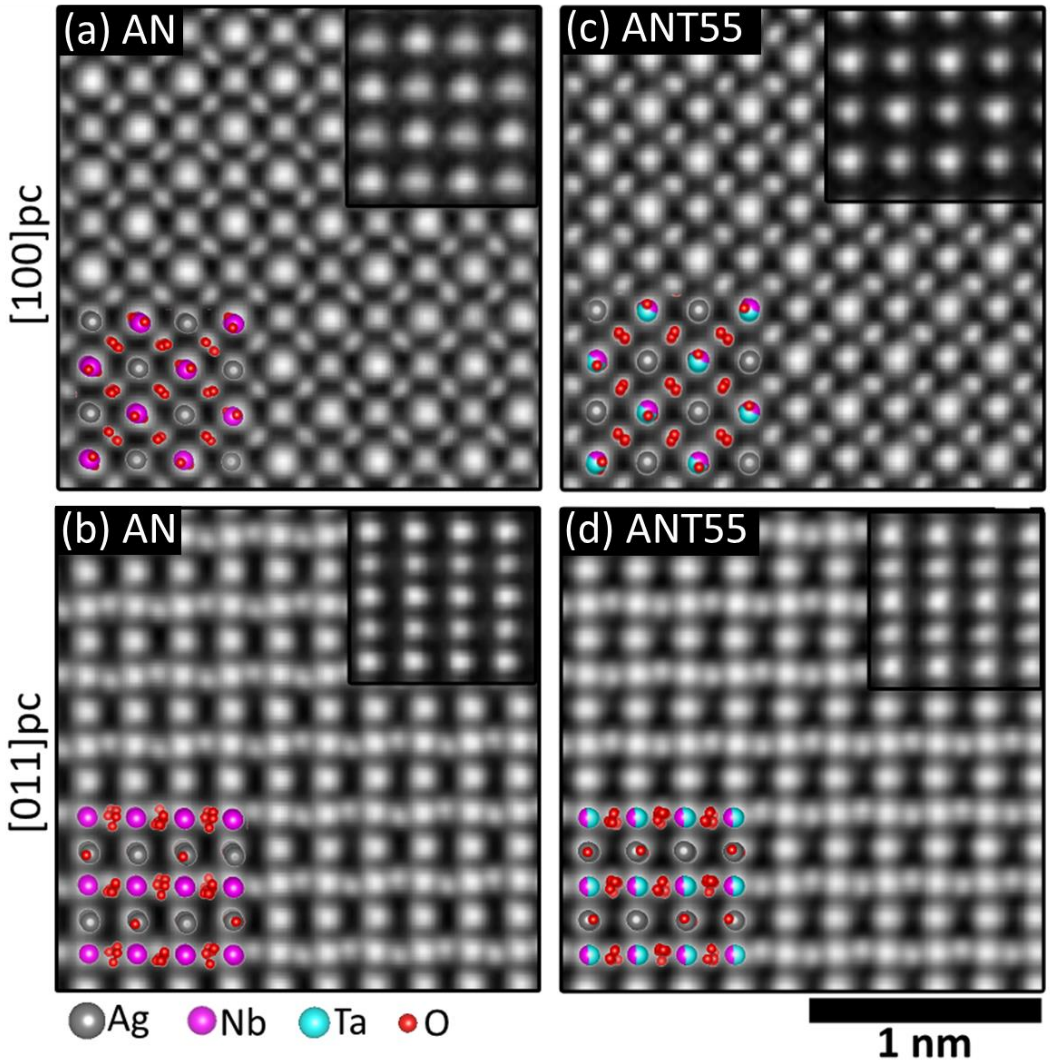

Supplementary Fig. 7 Integrated differential phase contrast (iDPC) images of AN and ANT55 samples. The atomic structure of AN samples oriented along the (a) $[100]_{pc}$ and (b) $[011]_{pc}$ zone axes. iDPC images showing the structure of ANT55 samples oriented along the (c) $[100]_{pc}$ and (d) $[011]_{pc}$ zone axes. Atomic structures generated from SXRD measurements for each composition are overlaid on their respective images. Inset on the top right of each image shows the annular dark-field image acquired simultaneously with the iDPC.

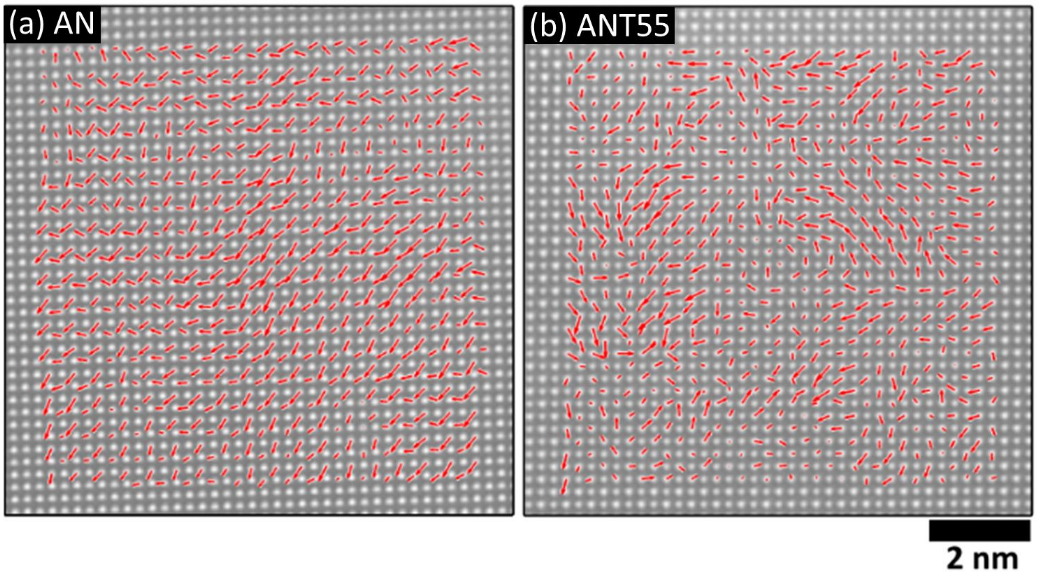

Supplementary Fig. 8 B sub-lattice cations displaced with respect to the A sub-lattice cations along the $[100]_{pc}$ zone axis. (a) AN and (b) ANT55 samples.
